# How does DICOM support big data management? Investigating its use in medical imaging community

**DOI:** 10.1186/s13244-021-01081-8

**Published:** 2021-11-08

**Authors:** Marco Aiello, Giuseppina Esposito, Giulio Pagliari, Pasquale Borrelli, Valentina Brancato, Marco Salvatore

**Affiliations:** 1 IRCCS SDN, Via Emanuele Gianturco 113, 80143 Naples, Italy; 2 Bio Check Up S.R.L, Naples, Italy

**Keywords:** DICOM, Big data, Data curation, COVID-19, Data analytics

## Abstract

The diagnostic imaging field is experiencing considerable growth, followed by increasing production of massive amounts of data. The lack of standardization and privacy concerns are considered the main barriers to big data capitalization. This work aims to verify whether the advanced features of the DICOM standard, beyond imaging data storage, are effectively used in research practice. This issue will be analyzed by investigating the publicly shared medical imaging databases and assessing how much the most common medical imaging software tools support DICOM in all its potential. Therefore, 100 public databases and ten medical imaging software tools were selected and examined using a systematic approach. In particular, the DICOM fields related to privacy, segmentation and reporting have been assessed in the selected database; software tools have been evaluated for reading and writing the same DICOM fields. From our analysis, less than a third of the databases examined use the DICOM format to record meaningful information to manage the images. Regarding software, the vast majority does not allow the management, reading and writing of some or all the DICOM fields. Surprisingly, if we observe chest computed tomography data sharing to address the COVID-19 emergency, there are only two datasets out of 12 released in DICOM format. Our work shows how the DICOM can potentially fully support big data management; however, further efforts are still needed from the scientific and technological community to promote the use of the existing standard, encouraging data sharing and interoperability for a concrete development of big data analytics.

## Key points

- Standardization is crucial for big data capitalization.
- DICOM supports key actions for imaging data management.
- The majority of shared research databases does not fully exploit DICOM format.
- Imaging software tools do not fully support DICOM advanced feature.
- There is need to fully promote DICOM in data sharing and software development.

## Introduction

The modern era is going through a rapid technological evolution, and we are witnessing the production of a huge amount of information that can be worthily enhanced with appropriate management and analysis. We are, in fact, in the so-called big data era, where the value of data can be the real engine of innovation [1].

The healthcare sector, where multi-sources patient information is routinely collected, is gaining volume and complexity. Among big data types, imaging data can be considered the largest in volume. In fact, it covers not only gigapixel images, such as tissues or organs at subcellular resolutions, but also metadata and quantitative measurements. For instance, neuroimaging is currently producing more than 10 petabytes of data every year with a staggering ninefold increase in data complexity (i.e., data acquisition modalities) over the last three decades [2, 3].

Therefore, the standardization of the medical imaging formats plays a crucial role in the effective exploitation of the data and subsequent clinical decision making [1].

The World Health Organization (WHO) recognizes the lack of standardization, together with privacy concerns, as the main barrier to big data exploitation [4]. The Digital Imaging and Communications in Medicine (DICOM) format is the current standard for storing and transmitting medical images, enabling the integration of medical imaging devices such as scanners, servers, workstations, printers, network hardware and picture archiving and communication systems (PACS) from multiple manufacturers [5]. DICOM encompasses raw imaging data and all the metadata related to the procedures of image acquisition and curation, including a series of processes such as de-identification of sensible data, annotation of regions of interest within the medical image, image enhancement or structured reporting.

In addition to DICOM, the most used formats designed for medical images are the analyzed format and its most recent version, Neuroimaging Informatics Technology Initiative (NIfTI), MetaImage (mhd) and nearly raw raster data (NRRD). They are mostly used for saving images for post-processing operations [6]. It is important to note, however, that DICOM remains the standard for the clinical management of images, which are routinely acquired in DICOM and eventually transformed into other formats. For this reason, our work does not focus on the already consolidated use of DICOM as a clinical standard but on the extension of the standard toward big data analytics, as a putative bridge between the clinic and research.

In fact, during the last years, the DICOM steering committee has carefully followed these needs by defining and extending the standard [7, 8]. Nevertheless, the simple definition of a standard is not enough to satisfy needs since the availability to users and the support with appropriate software tools are crucial.

This work aims to evaluate the effective implementation of the DICOM standard in the big data perspective and, in particular:

- To recognize and introduce how the DICOM standard effectively supports the current challenges in diagnostic imaging management and analytics.
- To verify whether the standard is implemented in clinical and research practice, investigating the public data shared by the research community and checking how much the standard is actually supported in the main software for management and processing of diagnostic images.

The scientific literature shows different initiatives that aim to evaluate the use of DICOM and related software tools for research purposes, in particular for the management of diagnostic data oriented to quantitative imaging [7, 9, 10] or for operations such as de-identification [11]; in this work, we intend to carry out a comprehensive evaluation that includes all the main data curation operations both for software tools and released datasets.

The next paragraphs deepen the introduction of this context and show the current support of the DICOM format in the diagnostic workflow.

### Big data workflow

Despite large strides in the introduction of PACS over the past few decades and the acceptance of the international DICOM standard for the storage and transfer of medical imaging data, there still remain significant barriers for the effective implementation of big data analytics on diagnostic imaging data.

Diagnostic images constitute a huge amount of data circulating in health care.

Unfortunately, medical data are often stored in qualitative reports, which do not allow recovering the images of interest as well as the related details. The clinical decision, in fact, is usually made on information deriving from several sources which may vary from the patient to the diagnostic systems but could not be stored in an appropriate and standardized way. Each of these single attributes could be considered as a fundamental element for the consequent definition of algorithms or, e.g., supervised or unsupervised models.

**Fig. 1:**
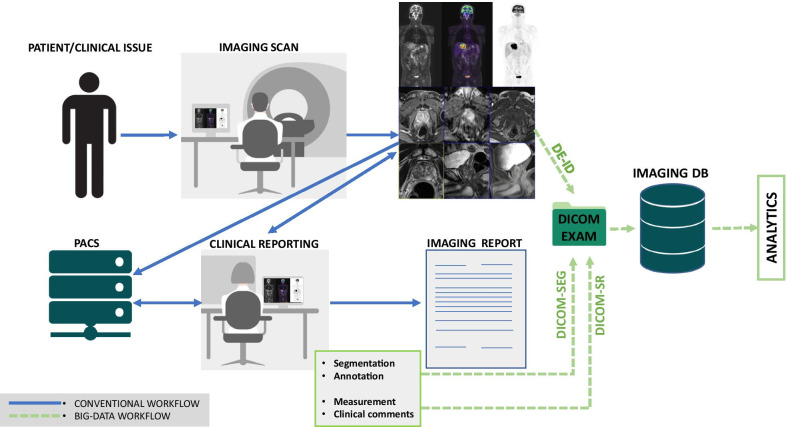
Conventional radiological workflow and collection of information for big data analytics using specific DICOM tags. Blue arrows refer to the radiological workflow; green arrows refer to data collection for big data analytics

Figure 1 shows a typical radiological workflow, in which the patient undergoes a diagnostic imaging examination. The results of the diagnostic procedure are elaborated by the instrumentation (e.g., computed tomography (CT) or magnetic resonance (MR) scan and stored in the DICOM format after the image formation procedure. Series, scans or reports are usually safely stored in the hospital's PACS and could be recalled by a radiologist or a clinician whenever needed. For instance, data retrieval is a fundamental step for reporting the result of the examination, identifying any potential pathologic condition and giving the patient or other practitioners the relevant indications. Many critical and significant elements may be lost during the conventional workflow because of the lack of standardization, the use of different formats or errors in the recording stage. We can recognize three fundamental actions, as shown in Fig. 1:

1. *De-identification/anonymization*: A DICOM file contains both the image and a large variety of data in the header. All of these elements can includeidentifiable information about the patient, the study and the institution. Sharing such sensitive data demands proper protection in order to ensure data safety and maintain patient privacy [12].
2. *Annotation/segmentation*: This phase includes all the operations of delineation, demarcation, localization and measurement of regions of interest (organs, lesions, suspicious or notable areas) within the diagnostic images [1]. As a final result, some quantitative data are reported with the images and could be used to build datasets for the development and validation of algorithms, models and the so-called semantic segmentation [13].
3. *Clinical reporting*: This operation includes the collection of data related to the patient's pathological state, before and after the diagnostic session. A report usually includes critical data such as the clinical outcomes, clinical characterization of the subjects and information about therapeutic treatments. Similar information has a huge potential in terms of data analysis but is often inaccessible due to the qualitative and descriptive nature of these documents.

All the information processed and recorded during these steps should be prepared as efficiently as possible: The resulting datasets should be analyzed readily and should not require additional curation steps. In fact, any additional procedure would be infeasible, especially considering large amounts of data.

The FAIR Guiding Principles [14, 15] state that good data management is “the key conduit leading to knowledge discovery and innovation, and to subsequent data and knowledge integration and reuse by the community after the data publication process.” FAIR, in fact, stands for findability, accessibility, interoperability and reusability, which are perceived as the four key factors affecting data quality.

It is important to note that, in the age of machine learning, “reusability” refers to reuse not only by humans but also by machines. Consequently, it is important to consider how to make data readable by machines in order to make best use of modern technologies.

### Privacy and de-identification

Privacy is one of the most discussed topics related to data collection, analysis and interpretation since, nowadays, these activities and their results are considered as a new business. Medical data may have several legitimate secondary uses, such as research projects or teaching, but it is strictly necessary to receive the informed consent from the patients. Another example is the development of decision support systems, which could be easily commercialized as soon as the results of the digital toolkit are considered as adequate. Additionally, most personal data should be removed securely, even if in many cases some data or a link to personal data must be kept. The increasing demand for predictive models, clinical decision support systems and data analysis has led to a situation where the research needs are often in conflict with privacy rules [16]. At this moment, there is no commonly accepted solution since the amount and type of personal data requested by researchers varies on a case-by-case basis and the choice of information to be kept, modified or deleted depends on the purposes and regulations to comply with.

To overcome these problems, governments and institutions have developed rules and regulations that bring together privacy, data and research purposes. To this aim, some of the most requested and used regulations that must be considered to use data for research purposes are:

- HIPAA, 1996 Health Insurance Portability and Liability Act (HIPAA),
- GDPR, the general data protection regulation (EU) 2016/679.

Specifically, the GDPR gives EU citizens or residents the right to request the deletion, modification or access of their data, while the HIPAA does not confer this right. The GDPR compliance affects all personal data, while the HIPAA is limited to the “Protected Health Information” (PHI), defined as information that can be used to directly or indirectly identify an individual in relation to his past, present or future health condition. In addition, the HIPAA contains the "Safe Harbor" method, which lists 18 identifiers of types to be removed or modified, whereas there are no explicit lists of the elements to be eliminated in the GDPR.

Pseudonymization and anonymization are different ways to perform de-identification. Specifically, following the ISO 25237:2017 standard, anonymization is defined as a “process by which personal data are irreversibly altered in such a way that a data subject can no longer be identified directly or indirectly, either by the data controller alone or in collaboration with any other party,” whereas pseudonymization is a “particular type of de-identification that both removes the association with a data subject and adds an association between a particular set of characteristics relating to the data subject and one or more pseudonyms.”

From these definitions, anonymization can be used in accordance with HIPAA and both anonymization and pseudonymization are considered as “data processing” under the GDPR. In the latter case, the right of access, modification and cancellation of the citizen's personal data must be guaranteed. However, as the anonymous data have no direct or indirect links to identify the original patient, any additional processing or processing performed on that dataset falls outside the scope of the GDPR.

The implementation of de-identification on clinical diagnostic examinations passes through the search, elimination and replacement of identifiers within the images and tags that describe them. Most DICOM objects contain demographic and associated medical images and information about the patient, which must be kept confidential or removed in case the tests are to be used for research purposes. As reported in the document “Security and Privacy in DICOM” [12], since 1999 the DICOM standard has included options to encrypt and protect data that move through the connections of network in response to the implementation of HIPAA and not in response to cybersecurity problems; furthermore, in 2001 DICOM extended the use of the CMS (Cryptographic Message Syntax) to encrypt DICOM data, allowing the encryption of the PHI of a DICOM object.

Profiles and options to address the removal and replacement of attributes within a DICOM Dataset are reported in the Annex E of DICOM PS3.15 2020d. Specifically, it contains a Basic Profile with several retain or clean options, and other implementations such as “In standard Compliance of IOD.” Each attribute can be either replaced, cleaned, removed or kept, depending on the confidentiality level and the importance of the identifier. The following tags should be added after the application of one of the de-identification profiles, to retain the history of metadata as in Table 1.

**Table 1:** The DICOM attributes to add after the application of the DICOM attribute confidentiality profiles

Attribute	Tag	Description	Type
Private data element characteristics sequence attribute	(0008, 0300)	Characteristics of private data elements within or referenced in the current SOP instance	3
Deidentification action sequence attribute	(0008, 0305)	Actions to be performed on element within the block that are not safe from identify leakage	3
Patient identity removed	(0012, 0062)	The true identity of the patient has been removed from the attributes and the pixel data	3
De-identification method	(0012, 0063)	A description or label of the mechanism or method use to remove the patient's identity. May be multi-valued if successive de-identification steps have been performed	1C
De-identification method code sequence	(0012, 0064)	A code describing the mechanism or method use to remove the patient's identity	1C
Burned in annotation attribute	(0028, 0301)	Indicates whether or not image contains sufficient burned in annotation to identify the patient and date the image was acquired	3
Recognizable visual features attribute	(0028, 0302)	Indicates whether or not the image contains sufficiently recognizable visual features to allow the image or a reconstruction from a set of images to identify the patient	3
Longitudinal temporal information modified	(0028, 0303)	Indicates whether or not the date and time attributes in the instance have been modified during de-identification	3
Encrypted attributes sequence attribute	(0400, 0500)	Sequence of items containing encrypted DICOM data	1C
Original attributes sequence attribute	(0400, 0561)	Sequence of items containing all attributes that were removed or replaced by other values in the top level dataset	3

Type 3: optional; Type 1C: conditional

There are various software tools to perform de-identification on DICOM images. Aryanto et al. [11] offer a complete overview and critical analysis.

It is very important to mention that simply removing or modifying metadata in DICOM images may not be sufficient to prevent re-identification of the subject. In fact, topograms for CT data and ultrasound images may have patient information burned into the pixel data [17]. To manage this critical issue, some specific studies and tools are available to the scientific community [18–21]. In addition, it is demonstrated that humans or specific software could identify individual subjects by reconstructing facial images contained in cranial MR or CT [22–24]. Further efforts are needed to develop reliable de-identification methods for medical images containing identifiable anatomical details, such as facial features. Moreover, the creation of specific DICOM attributes that encode this operation is expected to fully standardize the de-identification procedures.

### Annotation and segmentation

The key actions to extract fundamental features from medical images for both clinical decision making and research are annotation and segmentation. The annotation procedure focuses on labeling images with additional information useful for data detection, classification and grouping [25–28]. In particular, this routine allows to transform descriptive and qualitative image features in machine-readable data, thus making them suitable for automatic image analyses as supervised artificial intelligence (AI) methodologies [29]. Segmentation can be considered as a special case of annotation where one or more image areas are isolated in so-called regions of interest (ROI). Indeed, segmentation is based on drawing (manually, semi-automatically or fully automatically) a binary mask of pixels belonging to the ROI.

In the context of big data analytics, annotation and segmentation routines are fundamental processes for data description, sharing and analysis. Furthermore, the huge amount of data to be managed requires approaches able to codify and standardize the annotation data.

To address this issue, the National Electrical Manufacturers Association (NEMA) developed DICOM-RT, i.e., the first extension of the DICOM standard that could include information regarding annotations [30]. DICOM-RT was developed to specifically address the standardization of data deriving from radiotherapy (e.g., external beam, treatment planning, dose, radiotherapy images). In particular, five DICOM-RT objects were defined to manage: areas of significance (DICOM-RT Structure Set), transfer of treatment plans (DICOM-RT Plan), dose distribution of the radiation therapy plan (DICOM-RT Dose), radiotherapy images (DICOM-RT Image) and treatment session report (DICOM-RT Treatment Record). Segmentation information provided as ROIs was specifically included in DICOM-RT Structure Set object and, conditionally for data containing dose points or dose curves, in DICOM-RT Dose object.

More recently, DICOM-SEG [31] has been introduced. It is a dedicated modality in which the annotation routine is encoded as text (Table 2) and the positional information of the annotation is specified by a codified segmentation image (image data).

**Table 2:** Most pertinent/specific DICOM tags related to DICOM-SEG modality

Attribute	Tag	Description	Type
Image type	(0008, 0008)	Value reflecting if the image is primary or derived (value shall be 1 for DERIVED)	1
Instance number	(0020, 0013)	SOP instance number	1
Segmentation type	(0062, 0001)	Encoding properties of the segmentation (BINARY or FRACTIONAL)	1
Segmentation fractional type	(0062, 0010)	The meaning of fractional value (required for FRACTIONAL Segmentation type)	1C
Segments overlap	(0062, 0013)	Specify if one pixel can be in more than one segment	3
Segment sequence	(0062, 0002)	Description of the segment(s)	1
Segment number	(0062, 0004)	The number of the segment (unique)	1
Segment label	(0062, 0005)	User-defined label identifying the segment	1
Segment description	(0062, 0006)	User-defined description of the segment	3
Segment algorithm type	(0062, 0008)	Type of the algorithm used to generate the segment (AUTOMATIC, SEMIAUTOMATIC, MANUAL)	1
Segmented property category Segmented property type	(0062, 0003) (0062, 000F)	Sequence defining the specific property the segment represents	1
Definition source sequence	(0008, 1156)	Source sequence of the segment(s)	3
Segment algorithm name	(0062, 0009)	Name of the algorithm to generate the segment (required AUTOMATIC or SEMIAUTOMATIC in (0062,0008))	1C
Segmentation algorithm identification attribute	(0062, 0007)	A description of the segmentation algorithm	3

Type 1: required (valid value); Type 3: optional; Type 1C: conditional

It is worth mentioning that software to include segmentation/annotation information as DICOM-SEG modality was developed, thus allowing the management of such information in DICOM. In particular, dedicated software packages as DCMTK [32], ITK [33, 34], dcmqi [10] (built upon DCMTK and ITK) and pydicom-seg [35] are suitable for this purpose.

### Structured report

With the recent technical advances, the need to achieve full interoperability with the increasing amount of multi-modal patient data is arising. Structured reporting (SR) is becoming essential for clinical decision-making and research applications, including big data and machine learning.

SR aims to standardize both the format and lexicon used in radiology reports [36]. A definition for SR is set by describing three increasing levels of SR according to Weiss and Bolos [37]: The first and basic level consists of a structured format with paragraph and subheadings; the second is marked by a consistent organization with items reported in a certain order; and the third and more complex is characterized by the consistent use of dedicated lexicon and ontology.

The main reasons prompting to move from traditional free text reporting to standardized and structured reporting are summarized below and encompass both clinical and research considerations [38–40]. First of all, the use of checklist-style SR and standardized SR templates ensures that all relevant items for a particular examination are addressed. This may reduce diagnostic error, improve report clarity and quality and ensure consistent use of terminology across practices. Secondly, the use of standardized lexicon and structure prevents ambiguity and facilitates comparability of disease states, treatments and any type of clinical results. Even if this constitutes guidance for referring physicians, it should be highlighted that a simple comparison of the results is crucial for clinical purposes.

Finally, the capability of SR to include quantitative imaging biomarkers (radiomics) and parameters (e.g., laboratory results) might make SR as “Big-data container” leading not only to an integrated and precise clinical decision (e.g., diagnosis, treatment option) but also to a substantial support for modern clinical research. The standardized structure and vocabulary typical of SR can be well suitable to be analyzed by computers, thus facilitating data sharing (e.g., registries and biobanks) or data mining in research [38–41].

In summary, a wide adoption of SR is critical not only for communicating results to physicians or patients, but also for making diagnostic imaging data suitable by AI algorithms. Clinical decision-making and research applications in AI and big data in medical imaging heavily depend on data and standardization. One of the main challenges for the development of AI solutions for health care and radiology remains the unstructured nature of the data stored in electronic health records. In particular, radiological report data are often available only as unstructured narrative text [41–43].

The implementation of SR is complex and still scarce in clinical routine for several reasons. One of the biggest challenges in SR implementation is resistance to switch from the traditional narrative reporting to SR. Another issue concerns the risk of errors in case of improper use in clinical routine. Moreover, including unnecessary or irrelevant information in a template report may negatively impact the coherence of the report and the subsequent comprehension by referring physicians. Finally, the SR checklist schema may interfere with the radiologist’s reasoning and ability with a negative impact on the search pattern and visual attention. The so-called eye-dwell phenomenon may happen in case radiologists are more focused on the report template rather than the images. This may not only increase reporting time, but generate errors or missed findings [38–40].

Several steps were made by healthcare providers in order to overcome the above-mentioned limitations and encourage the use of uniform language and structure in radiology reporting, which are the basis for successfully implementing SR in clinical practice. For instance, RSNA developed RadLex, a standardized ontology of radiological terms in constant updating and developed starting from SNOMED-CT. RadLex can be used together with popular medical lexicons such as SNOMED-CT, ICD-10, CPT and BrainInfo [44]. Moreover, RSNA started the so-called Reporting Initiative with the aim of developing and providing vendor-neutral reporting templates [45]. This led to the publication of the Management of Radiology Report Templates profile by IHE, which extensively describes the concepts and technical details for interoperable, standardized and structured report templates [46].

Since an essential requirement for the successful implementation of SR is to respect the current radiology workflow, the DICOM standard plays a key role [5]. Given its potentiality, NEMA introduced the DICOM-SR which defines the syntax and semantics of structured and standardized diagnostic reports. An exhaustive description of DICOM-SR can be found in Clunie’s work [47]. Briefly, like a DICOM image, the DICOM-SR has a header, which encodes the information of the patient and study identification, and a content, that instead is responsible for the coding of the report itself. The information elements in the report are hierarchically connected in a tree model, identifying the Sources and Targets Nodes and their relationships. Each element has a name and a value, forming the pairs Name-Value [48, 49]. DICOM-SR contains text with links to other data such as images, waveforms and spatial or temporal coordinates. Although DICOM-SR is not as widespread as DICOM for digital images, its use has many advantages [50, 51]. DICOM-SR documents can be stored and sent along with the images belonging to the same study in PACS. In addition, DICOM-SR supports unified lexicons such as RadLex, ICD-10 and SNOMED. Finally, DICOM-SR templates have been defined to constrain the possible structures and to provide the basic codes that can be used to encode specific reports [50]. Specifically, a DICOM-SR template is applied to the document content to harmonize its structure. Each template is assigned to an unique template identifier (TID) with a related name and is specified by a table where each line corresponds to a so-called node with defined content item or indicates another template to be included in the SR document [48]. A specific limitation of DICOM-SR is that even if it provides a data structure which embeds structured reports in a standard “container” that can be read across different software applications, it does not define how the content should be structured or standardized.

**Fig. 2:**
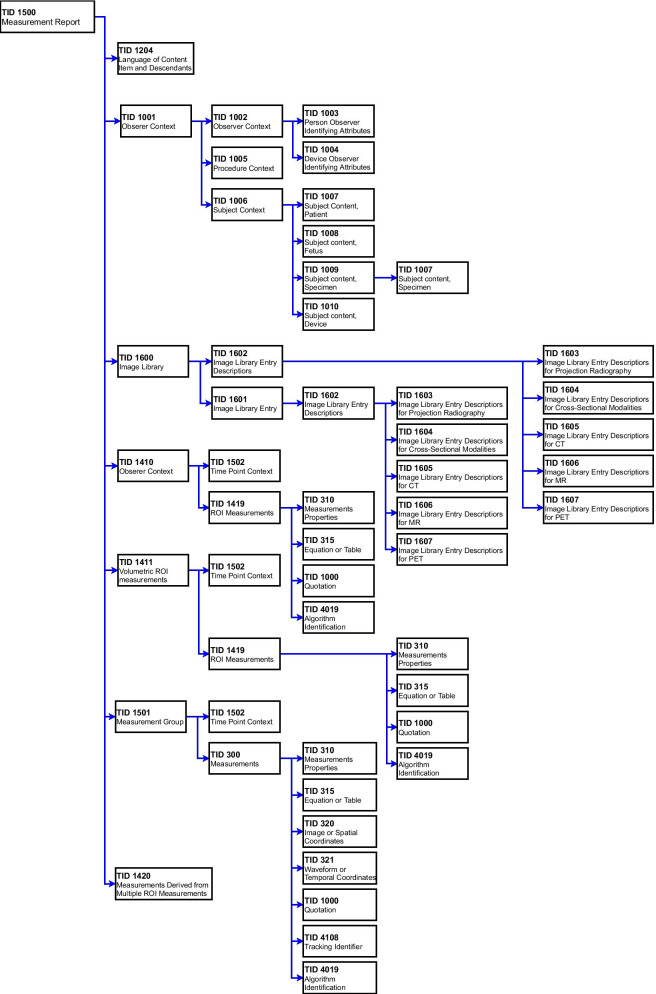
DICOM SR measurement report template structure (template ID 1500) and its sub-templates (from DICOM PS3.21). TID: template ID, MR, magnetic resonance; CT, computed tomography; PET, positron emission tomography

Table 3 and Fig. 2 show the definition of the DICOM-SR Template for a Measurement Report and its sub-templates (TID 1500 from DICOM PS3.16 and PS3.21).

**Table 3:** DICOM SR template for measurement report, template ID 1500 (from DICOM PS3.16)

	NL	Rel with parent	VT	Concept name	VM	RT	Cond	Value set constraint
1	>		CONTAINER	DCID 7021 “Measurement report document titles”	1	M		Root node
2	>	HAS CONCEPT MOD	INCLUDE	DTID 1204 “Language of content item and descendants”	1	U		
3	>	HAS OBS CONTEXT	INCLUDE	DTID 1001 “Observation context”	1	M		
4	>	HAS CONCEPT MOD	CODE	EV (121,058, DCM, “Procedure reported”)	1-n	U		BCID 100 “Quantitative Diagnostic Imaging Procedures”
5	>	CONTAINS	INCLUDE	DTID 1600 “Image library”	1	U		
6	>	CONTAINS	CONTAINER	EV (126,010, DCM, “Imaging measurements”)	1	C	IF row 10 and 12 are absent	
6b	>>	HAS CONCEPT MOD	INCLUDE	DTID 4019 “Algorithm identification”	1	U		
7	>>	CONTAINS	INCLUDE	DTID 1410 “Planar ROI measurements and qualitative evaluations”	1-n	U		$Measurement = BCID 218 “Quantitative Image Features” $Units = BCID 7181 “Abstract Multi-dimensional Image Model Component Units” $Derivation = BCID 7464 “General Region of Interest Measurement Modifiers” $Method = BCID 6147 “Response Criteria” $QualModType = BCID 210 “Qualitative Evaluation Modifier Types” $QualModValue = BCID 211 “Qualitative Evaluation Modifier Values”
8	>>	CONTAINS	INCLUDE	DTID 1411 “Volumetric ROI measurements and qualitative evaluations”	1-n	U		$Measurement = BCID 218 “Quantitative Image Features” $Units = BCID 7181 “Abstract Multi-dimensional Image Model Component Units” $Derivation = BCID 7464 “General Region of Interest Measurement Modifiers” $Method = BCID 6147 “Response Criteria” $QualModType = BCID 210 “Qualitative Evaluation Modifier Types” $QualModValue = BCID 211 “Qualitative Evaluation Modifier Values”
9	>>	CONTAINS	INCLUDE	DTID 1501 “Measurement and qualitative evaluation group”	1-n	U		$Measurement = BCID 218 “Quantitative Image Features” $ImagePurpose = BCID 7551 “Generic Purpose of Reference to Images and Coordinates in Measurements” $Units = BCID 7181 “Abstract Multi-dimensional Image Model Component Units” $Derivation = BCID 7464 “General Region of Interest Measurement Modifiers” $Method = BCID 6147 “Response Criteria” $QualModType = BCID 210 “Qualitative Evaluation Modifier Types” $QualModValue = BCID 211 “Qualitative Evaluation Modifier Values”
10	>	CONTAINS	CONTAINER	EV (126,011, DCM, “Derived imaging measurements”)	1	C	IF row 6 and 12 are absent	
10b	>>	HAS CONCEPT MOD	INCLUDE	DTID 4019 “Algorithm identification”	1	U		
11	>>	CONTAINS	INCLUDE	DTID 1420 “Measurements derived from multiple ROI measurements”	1-n	U		
12	>	CONTAINS	CONTAINER	EV (C0034375, UMLS, “Qualitative evaluations”)	1	C	IF row 6 and 12 are absent	
12b	>>	HAS CONCEPT MOD	INCLUDE	DTID 4019 “Algorithm identification”	1	U		
13	>>	CONTAINS	CODE		1-n	U		
13b	> >	HAS CONCEPT MOD	CODE	BCID 210 “Qualitative evaluation modifier Types”	1-n	U		BCID 211 “Qualitative Evaluation Modifier Values”
14	>>	CONTAINS	TEXT		1-n	U		

NL, nesting level, defining the tree structure and depth; VT, value type; BCID, baseline context group identifier; DCID, defined context group identifier; VM, value multiplicity, defining if a tree node may appear only once or can be repeated; RT, requirement type, defining if a tree node is mandatory or optional; EV, enumerated value

Similar to DICOM-SEG, several efforts have been made in software development supporting the management of structured reports in DICOM standard [10, 32–34].

## Materials and methods

To evaluate the degree of effective use of the DICOM standard in the previously detailed actions (i.e., de-identification/anonymization, segmentation/annotation and clinical reporting), two different experiments have been designed and will be described in the following paragraphs. The first explores the DICOM coding of information in the main databases shared by the scientific community, whereas the second investigates and evaluates the possibility of managing DICOM fields with a systematic selection of DICOM visualization software.

### Imaging database evaluation

The databases included in our benchmark have been collected by a quasi-systematic internet survey, starting from four well-known lists of public repositories [52–55].

The study of databases aims to identify the type of information released and to evaluate its standardization. In particular, in this analysis we intend to verify whether the DICOM standard is used to collect, where possible, the information available.

Each database has been evaluated considering its modality of access and several key features, described hereafter. Furthermore, we performed a three-step review of the available repositories, in order to select which of them were suitable for this project. The datasets released up to March 1, 2021, were included. The detailed description of the database selection criteria is as follows:

Step 1:

- *Access*: We looked for open or public access. In particular, repositories which required an application with a project, or private datasets were not considered.
- *Format*: Only datasets providing imaging scans in DICOM format were selected.
- *Species*: We considered only humans, whereas there were few databases with rodents, small animals or phantom images.

Step 2:

Among selected databases providing images in DICOM format, we excluded those not including any additional information than the acquired images. Specifically, we considered the repositories having at least one of the following additional resources, not necessarily provided in DICOM format:

- annotations/segmentations;
- radiologist reports;
- any clinical information.

In this step, the databases that provided clinical information, in any format, were included. The latter criterion was adopted since, in this step, we aimed to verify how many databases had sufficient information to reconstruct dedicated DICOM-SR. Specifically, we considered this condition verified in the following cases:

1. traceability of the referenced images;
2. clinical information related to single imaging modality.

Step 3:

From the repositories identified in step 2, we selected databases that provided additional information according to the DICOM standard. In particular, we excluded all databases not including at least DICOM-SEG or DICOM-SR. In the case of TCIA repository, we used the TCIA portal (https://nbia.cancerimagingarchive.net/nbia-search) for selection and filtering of the collections. Moreover, the collections containing the TCIA third-party analysis results provided in DICOM-SEG or DICOM-SR modalities were also included. In particular, the aforementioned derived data corresponded to contributions generated by researchers who were not part of the group which originally submitted the related TCIA collection.

In order to test the actual presence of available DICOM images, and any associated additional information in DICOM format, each dataset was inspected in its entire content. Specifically, the first patient folder containing DICOM images was opened by using PostDicom software (https://www.postdicom.com/), which allows the reading of DICOM series. Exploiting PostDicom's ability of reading information encoded in both the DICOM-SEG and DICOM-SR, the same procedure was performed for the associated additional information in DICOM format (if present).

Furthermore, to complement the analysis, it was considered appropriate to investigate the sharing of imaging data during the spread of coronavirus disease 2019 (COVID-19). The COVID-19 pandemic required a joint effort for the urgent development of tools and data sharing to better face the emergency. In this situation, diagnostic imaging also plays a crucial role since the virus can generate a viral lung infection with a typical pattern in the chest computed tomography (CT), such as ground glass opacity, crazy-paving pattern and consolidation [56].

In particular, initiatives for the development of automatic diagnostic support tools based on AI techniques for diagnosis and clinical prediction have proliferated [57]. For the development of these tools, it is essential to make available large and curated datasets that include, in addition to imaging scans, information relating to the segmentation of regions of interest (i.e., lesions generated by viral pneumonia) and information related to the clinical status of the subject and the clinical outcome [58–61]. It is clear that, in this context, the use of standards plays a key role and, therefore, the analysis of CT COVID-19 datasets sharing deserves particular attention.

For this purpose, publicly accessible datasets including chest CT images with COVID-19 lesions, available at the first pandemic year (up to March 2021), have been identified in leading scientific research repositories. For each dataset, a sample of the data was downloaded to verify the format used for the release of CT scans and associated segmentations and clinical information.

### Imaging software evaluation

In this section, we specifically consider the analysis of the software tools suitable for the diagnostic workflow. For this reason, the tools allowing at least the direct visualization of the DICOM images have been focused on, and their ability to manage the other data curation actions has been analyzed.

The software and tools included in our benchmark have been collected by a quasi-systematic Internet survey using the I Do Imaging (IDI) [62] initiative. Moreover, all software tools listed in the DICOM/PACS viewer webpage of Radiology Cafè [63] have been included to account for relevant software tools intended for clinical use.

The first includes all free software tools released and reviewed for research purposes, whereas the latter includes all software tested by a consultant radiologist and thought to represent the best currently available online, including core functionalities required by a radiologist for reviewing studies and/or teaching. In order to meet our evaluation criteria, the IDI database has been filtered for DICOM support, display of images and rank greater than four stars. Note that we have not included software or tools with no rating. Each software has been evaluated according to the following inclusion criteria:

- *DICOM format*: We have considered only software tools that could at least read a DICOM series.
- *Display*: This is a mandatory requirement, each software should, at least, display the image contained in a DICOM series.
- *License*: We considered free or open-source software. Commercial software or tools were included only if a free trial version was available.

After selecting software tools that fulfilled the inclusion criteria, we considered the following features for the software evaluation procedure:

- *De-identification*: Software that could modify a DICOM header, e.g., editing or removing tag values and save back all the de-identified personal information;
- *Segmentation and annotation*: Software and tools that could read and/or write a DICOM file with segmentations or annotations, using the dedicated DICOM tags;
- *Structured report*: Reading and writing DICOM structured reports, i.e., enclosed documents or data.

To evaluate if a specific software was able to read a DICOM-SR file, we checked if DICOM-SR content was successfully read by the software and displayed to the operator.

In order to test the functionality of the software selected in the management of the DICOM fields, specific “probe” datasets have been created. Two exemplary DICOM publicly available folders, specifically downloaded from TCIA Prostate-diagnosis [64] and CMET-MRhead [65], served for this scope. For the segmentation and annotation functionalities, the data were already embedded as DICOM-SEG modality in CMET-MRhead repository. Conversely, since NRRD format was used for these data types in Prostate-Diagnosis repository, dcmqi tool [10] was used to convert NRRD segmentations to DICOM-SEG. To generate DICOM-SR from the probe datasets, the template TID1500 “Measurement Report” was used (from DICOM PS3.16, http://dicom.nema.org/medical/dicom/current/output/html/part16.html#sect_TID_1500). In particular, for both probe datasets, basic measurements (e.g., mean, standard deviation, median, range, volume) were evaluated on the original DICOM images by means of the associated DICOM-SEG and encapsulated in DICOM-SR by using the dcmqi package [10].

All software and tools ran on a Windows V10 operating system, except for the Horos DICOM Viewer that was tested on Mac OS Catalina V10.15.6.

## Results

### Imaging database evaluation

Regarding the selection of databases to be analyzed, 210 databases were identified after the quasi-systematic web survey. 100 repositories were collected after the first selection criteria (step 1), among which 83 databases were selected by applying the criteria of step 2.

**Fig. 3:**
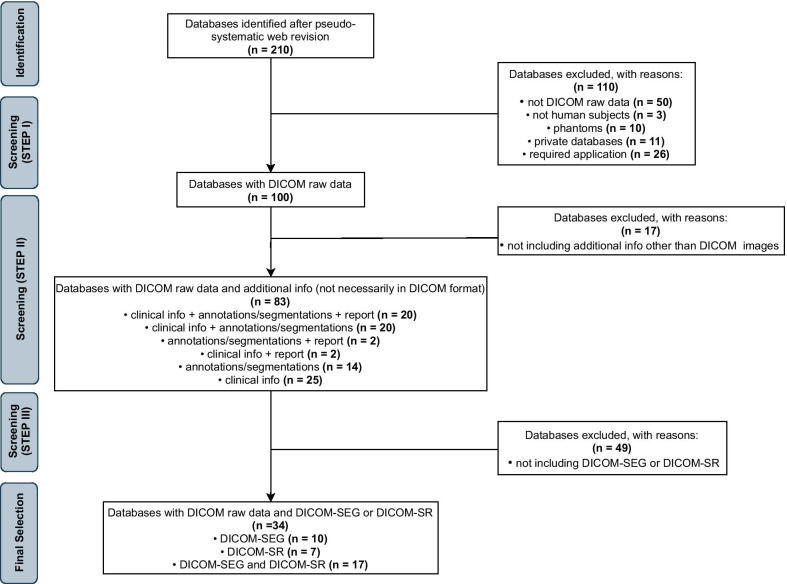
Flow diagram describing the process of imaging database evaluation and selection. DICOM, Digital Imaging and Communications in Medicine; DICOM-SEG, DICOM segmentation object; DICOM-SR, DICOM structured report object

In step 3, we identified which of the 83 detected databases provided additional information according to the DICOM standard. In particular, we excluded all databases not including at least DICOM-SEG or DICOM-SR. After the exclusion of 49 items, 34 datasets were finally selected (Fig. 3).

Table 4 shows the 83 databases identified after step 2, with lines colored in bold corresponding to the 34 datasets selected after applying step 3 criteria, including their characteristics (e.g., dataset name, collection, pathology) and additional information other than DICOM images (e.g., CI, clinical info; A/S, annotations/segmentations; CR, clinical report). Focusing on the seventh, eighth and ninth columns (CI, A/S, CR), it is evident that, respectively:

- No clinical information, when available, is reported in DICOM format. Of the 67 databases including clinical information, only 29 provided clinical information that are sufficient to reconstruct DICOM-SR;
- Twenty-seven out of 56 databases that contain the definition of regions of interest use the DICOM-SEG format.
- Twenty-four datasets providing clinical reports, out of 83, use the DICOM-SR format. Furthermore, it was verified that all datasets comply with the correct DICOM format for data de-identification.

**Table 4:** Selected databases (*n* = 83) providing additional information other than DICOM images

Dataset name	Dataset collection	Pathology	Region	Modality	Number of samples	CI	A/S	CR	TCIA analysis results
PNEUMONIA	RSNA	Pneumonia	Lung	RX	30,000	N	Y (JSON)	N	
CT Lymph Nodes	TCIA	Lymphadenopathy	Abdomen, mediastinum	CT	176	N	Y (NIfTI)	N	
Pancreas-CT	TCIA	Healthy controls	Pancreas	CT	82	N	Y (NIfTI)	N	
Prostate-3 T	TCIA	Prostate cancer	Prostate	MR	64	N	Y (.mhd)	N	
**RIDER Lung CT**	**TCIA**	**Lung cancer**	**Chest**	**CT**	**32**	**N**	Y (XLS), Y^*a^**, Y**^**R**^	**N**	[78, 79]
**Brain-Tumor-Progression**	**TCIA**	**Brain cancer**	**Brain**	**MR**	**20**	**N**	Y^*^	**N**	
**CBIS-DDSM**	**TCIA**	**Breast cancer**	**Breast**	**MG**	**1566**	**N**	Y^*^	**N**	
**QIN LUNG CT**	**TCIA**	**Non-small cell lung cancer**	**Lung**	**CT**	**47**	**N**	**Y**^**a**^ (NIfTI), Y^*a^	**N**	[78–82]
4D-Lung	TCIA	Non-small cell lung cancer	Lung	CT	20	N	Y^R^	N	
AAPM RT-MAC Grand Challenge 2019	TCIA	Head and neck cancer	Head–neck	MR	55	N	Y^R^	N	
Head–Neck Cetuximab (RTOG 0522)	TCIA	Head and neck carcinomas	Head–neck	CT, PT	111	N	Y^R^	N	
LCTSC	TCIA	Lung cancer	Lung	CT	60	N	Y^R^	N	
MRI-DIR	TCIA	Squamous cell carcinoma	Head and Neck	MR, CT	9	N	Y^R^	N	
**Anti-PD-1 Lung**	**TCIA**	**Lung cancer**	**Lung**	**CT, PT, SC**	**46**	**N**	Y^*a^	Y^*a^	[83]
**Anti-PD-1_MELANOMA**	**TCIA**	**Melanoma**	**Skin**	**CT, MR, PT**	**47**	**N**	Y^*a^	Y^*a^	[83]
CT Colonography (ACRIN 6664)	TCIA	Colon cancer	Colon	CT	825	Y^b^	N	N	
LDCT-and-Projection-data	TCIA	Various	Head, chest, abdomen	CT	300	Y^b^	N	N	
NSCLC-Radiomics-Genomics	TCIA	Lung cancer	Lung	CT	89	Y^b^	N	N	
REMBRANDT	TCIA	Low- and high-grade glioma	Brain	MR	130	Y^b^	N	N	
Acrin-FLT-Breast (ACRIN 6688)	TCIA	Breast cancer	Breast	PET, CT, OT	83	Y	N	N	
Acrin-FMISO-Brain (ACRIN 6684)	TCIA	Glioblastoma	Brain	CT, MR, PT	45	Y	N	N	
ACRIN-NSCLC-FDG-PET (ACRIN 6668)	TCIA	Non-small cell lung cancer	Lung	PT, CT, MR, CR, DX, SC, NM	242	Y	N	N	
CPTAC-CM	TCIA	Cutaneous melanoma	Skin	MR, CT, CR, PT, pathology	94	Y	N	N	
CPTAC-LSCC	TCIA	Squamous cell carcinoma	Lung	CT, CR, DX, NM, PT, pathology	212	Y	N	N	
CPTAC-LUAD	TCIA	Adenocarcinoma	Lung	CT, MR, PT, CR, pathology	244	Y	N	N	
TCGA-CESC	TCIA	Cervical squamous cell carcinoma and endocervical adenocarcinoma	Cervix	MR, pathology	54	Y^b^	N	N	
TCGA-ESCA	TCIA	Esophageal carcinoma	Esophagus	CT, pathology	16	Y^b^	N	N	
TCGA-KICH	TCIA	Kidney chromophobe	Kidney	CT, MR, pathology	15	Y	N	N	
TCGA-KIRP	TCIA	Kidney renal papillary cell carcinoma	Renal	CT, MR, PT, pathology	33	Y	N	N	
TCGA-PRAD	TCIA	Prostate cancer	Prostate	CT, PT, MR, Pathology	14	Y	N	N	
TCGA-READ	TCIA	Rectum adenocarcinoma	Rectum	CT, MR, pathology	3	Y	N	N	
TCGA-SARC	TCIA	Sarcomas	Chest, abdomen, pelvis, leg, TSpine	CT, MR, pathology	5	Y	N	N	
TCGA-STAD	TCIA	Stomach adenocarcinoma	Stomach	CT, pathology	46	Y^b^	N	N	
TCGA-THCA	TCIA	Thyroid cancer	Thyroid	CT, PT, pathology	6	Y	N	N	
LGG-1p19qDeletion	TCIA	Low grade glioma	Brain	MR	159	Y^b^	Y (NIfTI)	N	
Lung Fused-CT-Pathology	TCIA	Lung cancer	Lung	CT, pathology	6	Y^b^	N	N	
LungCT-Diagnosis	TCIA	Lung cancer	Lung	CT	61	Y^b^	Y (XLS), Y^a^ (NIfTI)	N	[80–82]
Prostate-Diagnosis	TCIA	Prostate cancer	Prostate	MR	92	Y^b^	Y (.NRRD)	N	
PROSTATEx	TCIA	Prostate cancer	Prostate	MR	346	Y^b^	Y (CSV)	N	
SPIE-AAPM Lung CT Challenge	TCIA	Lung cancer	Lung	CT	70	Y^b^	Y (XLS)	N	
**Head-Neck-Radiomics-HN1**	**TCIA**	**Head and neck cancer**	**Head–neck**	**CT, PT, SEG**	**137**	**Y**^**b**^	Y^*^,Y^**R**^	**N**	
**NSCLC-Radiomics**	**TCIA**	**Lung cancer**	**Lung**	**CT, SEG**	**422**	**Y**^**b**^	Y^*^, Y^**a**^** (NIfTI), Y**^**R**^	**N**	[84–86]
**NSCLC-Radiomics-Interobserver1**	**TCIA**	**Non-small cell lung cancer**	**Lung**	**CT, SEG**	**22**	**Y**^**b**^	Y^*^, Y^**R**^	**N**	
**TCGA-GBM**	**TCIA**	**Glioblastoma multiforme**	**Brain**	**MR, CT, DX, pathology**	**262**	**Y**	Y^*a^**, Y**^**a**^** (NIfTI, XML)**	**N**	[87–90]
**TCGA-LGG**	**TCIA**	**Low-grade glioma**	**Brain**	**MR, CT, pathology**	**199**	**Y**	Y^*a^**, Y**^**a**^** (NIfTI)**	**N**	[88–90]
Head-Neck-PET-CT	TCIA	Head and neck cancer	Head–neck	PT, CT	298	Y^b^	Y^R^	N	
HNSCC	TCIA	Head and neck squamous cell carcinoma	Head–neck	CT, PT, MR	627	Y	Y^R^	N	
HNSCC-3DCT-RT	TCIA	Head and neck squamous cell carcinoma	Head–neck	CT, DICOM-RT, RTDOSE	31	Y^b^	Y^R^	N	
OPC-Radiomics	TCIA	Oropharyngeal	Head-and-neck	CT, DICOM-RT, Clinical	606	Y^b^	Y^R^	N	
Soft-tissue-Sarcoma	TCIA	Soft-tissue sarcoma	Extremities	FDG-PET/CT, MR	51	Y	Y^R^	N	
**CPTAC-CCRCC**	**TCIA**	**Clear cell carcinoma**	**Kidney**	**CT, MR, pathology**	**222**	**Y**	Y^*a^	Y^*a^	[83]
**CPTAC-GBM**	**TCIA**	**Glioblastoma multiforme**	**Brain**	**CT, CR, SC, MR, pathology**	**189**	**Y**	Y^*a^	Y^*a^	[83]
**CPTAC-HNSCC**	**TCIA**	**Head and neck cancer**	**Head–neck**	**CT, MR, SC, pathology**	**112**	**Y**	Y^*a^	Y^*a^	[83]
**CPTAC-PDA**	**TCIA**	**Ductal adenocarcinoma**	**Pancreas**	**CT, MR, DX, PT, XA, CR, US, pathology**	**168**	**Y**	Y^*a^	Y^*a^	[83]
**TCGA-BLCA**	**TCIA**	**Bladder endothelial carcinoma**	**Bladder**	**CT, CR, MR, PT, DX, pathology**	**120**	**Y**	Y^*a^	Y^*a^	[83]
**TCGA-COAD**	**TCIA**	**Colon adenocarcinoma**	**Colon**	**CT, pathology**	**25**	**Y**	Y^*a^	Y^*a^	[83]
**TCGA-LUSC**	**TCIA**	**Lung squamous cell carcinoma**	**Lung**	**CT, NM, PT, pathology**	**37**	**Y**	Y^*a^	Y^*a^	[83]
**TCGA-UCEC**	**TCIA**	**Uterine corpus endometrial carcinoma**	**Uterus**	**CT, CR, MR, PT, pathology**	**65**	**Y**	Y^*a^	Y^*a^	[83]
**Breast-MRI-NACT-Pilot**	**TCIA**	**Breast cancer**	**Breast**	**MR, SEG**	**64**	**Y**^**b**^	Y^*^	Y^*a^	[91]
**C4KC-KiTS**	**TCIA**	**Kidney cancer**	**Kidney**	**CT, SEG**	**210**	**Y**^**b**^	Y^*^	**N**	
**ISPY1 (ACRIN 6657)**	**TCIA**	**Breast cancer**	**Breast**	**MR, SEG**	**222**	**Y**^**b**^	Y^*^	Y^*a^	[91]
**NSCLC-Radiogenomics**	**TCIA**	**Non-small cell lung cancer**	**Chest**	**PT, CT, SEG, SR**	**211**	**Y**^**b**^	Y (XML), Y^*^, Y^*a^	Y^*a^	[83]
**TCGA-HNSC**	**TCIA**	**Head and neck squamous cell carcinoma**	**Head-neck**	**CT, MR, PT, Pathology**	**227**	**Y**	Y^*a^**, Y**^**R**^	Y^*a^	[83]
**LIDC-IDRI**	**TCIA**	**Lung cancer**	**Chest**	**CT, CR, DX**	**1010**	**Y**^**b**^	Y (XML), Y^*a^	Y^*a^	[78, 79, 92–95]
**QIN-HeadNeck**	**TCIA**	**Head and neck carcinomas**	**Head-neck**	**PT, CT, SR, SEG, RWV**	**156**	**Y**^**b**^	Y^*^	Y^*^	
**CPTAC-UCEC**	**TCIA**	**Corpus endometrial carcinoma**	**Uterus**	**CT, MR, PT, CR, DX, SR, pathology**	**250**	**Y**	Y^*a^	Y^*^ (incomplete SR), Y^*a^	[83]
**TCGA-KIRC**	**TCIA**	**Kidney renal clear cell carcinoma**	**Renal**	**CT, MR, CR, pathology**	**267**	**Y**	**Y**^**a**^** (XLS)**	Y^*a^	[96]
**TCGA-BRCA**	**TCIA**	**Breast cancer**	**Breast**	**MR, MG, pathology**	**139**	**Y**	**Y**^**a**^** (XLS)**	Y^*a^	[91, 97–102]
**TCGA-LIHC**	**TCIA**	**Liver hepatocellular carcinoma**	**Liver**	**MR, CT, PT, pathology**	**97**	**Y**	**Y**^**a**^** (XML)**	Y^*a^	[96]
**TCGA-LUAD**	**TCIA**	**Lung adenocarcinoma**	**Chest**	**CT, PT, NM, pathology**	**69**	**Y**	**Y**^**a**^** (XLS)**	Y^*a^	[96]
**TCGA-OV**	**TCIA**	**Ovarian serous cystadenocarcinoma**	**Ovary**	**CT, MR, pathology**	**143**	**Y**	**Y**^**a**^** (XLS)**	Y^*a^	[96]
**CPTAC-SAR**	**TCIA**	**Sarcomas**	**Various (11 locations)**	**CT, MR, PT, SR, pathology**	**94**	**Y**	**N**	Y^*^ (incomplete SR)	
**Breast Diagnosis**	**TCIA**	**Breast cancer**	**Breast**	**MR, PT, CT, MG**	**88**	**Y**^**b**^	**N**	Y (.XLS), Y^*a^	[91]
COVID-19-AR	TCIA	COVID-19	Chest	CT, CR, DX	105	Y	N	N	
MIDRC-RICORD-1a	TCIA	COVID-19	Chest	CT	110	Y^b^	Y (.JSON)	N	
MIDRC-RICORD-1b	TCIA	COVID-19	Chest	CT	117	Y^b^	N	N	
MIDRC-RICORD-1c	TCIA	COVID-19	Chest	CR, DX	361	Y^b^	Y (.JSON)	N	
ELCAP Public Lung Image Database	NA	Lung cancer	Lung	CT	50	N	Y (.CSV)	N	
I2CVB Prostate	NA	Prostate cancer	Prostate	MR	12	Y	N	N	
MIMBCD-UI UTA4	MIMBCD	Breast cancer	Breast	US, MG, MRI	3	Y	N	N	
MIMBCD-UI UTA7	MIMBCD	Breast cancer	Breast	US, MG	6	Y	Y (.JSON)	N	
MIMBCD-UI UTA10	MIMBCD	Breast cancer	Breast	US, MG	6	Y	Y (.JSON)	N	
eNKI_RS_TRT	NKI_RS	No	Brain	MRI	24	Y	N	N	

TCIA, The Cancer Imaging Archive; CT, computed tomography; MR, magnetic resonance; PT (or PET), positron emission tomography; SR, structured report; CR, computed radiography; MG, mammography; DX, digital radiography; XA, X-ray angiography; SC, secondary capture; NM, nuclear medicine; OT, other modality; RWV, real-world value; CI, clinical info; No, number of cases; A/S, annotations/segmentations; CR, clinical report; JSON, JavaScript object notation; NIfTI, Neuroimaging Informatics Technology Initiative; CSV, comma separated value; XLS, excel spreadsheet; XML, extensible markup language; NRRD, nearly raw raster data; mhd, MetaImage; Y, yes; N, no; NA, not applicable

Lines with bold correspond to the finally selected datasets (*n* = 34)

^*^Additional information provided according to DICOM format (DICOM-SEG for annotations/segmentations and DICOM-SR for clinical reports)

^a^Annotations/segmentations and/or clinical reports provided by researchers who were not part of the group which originally submitted the related TCIA collection, with related references reported in “TCIA analysis results” column

^b^Clinical info supposed to be enough to reconstruct DICOM-SR (namely if related imaging series is provided and/or if the clinical information refers to a single imaging modality)

^R^Annotations/segmentations provided as DICOM-RT

Of note, the highest result variability was found for A/S data (eighth column in Table 4). Indeed, excluding databases that provided segmentation or annotation in DICOM-SEG, ten databases released segmentation data in DICOM-RT, ten databases in image format (eight in NIfTI, one in mhd and one in NRRD data type), nine stored in tabular format (XLS and CSV data type) and nine stored in structured data format (four in XML and five in JSON data type). Except for image format, the remaining data formats require dedicated routines to manage A/S information by associating file content to positional information.

Focusing on the COVID-19 databases, 12 datasets have been identified [66–77]. Of note, we found that only two of the reviewed COVID-19 datasets were released in DICOM format [76, 77].

Five datasets use a non-specific image format for the medical domain as portable network graphics [66–68], tagged image file format [69] and hierarchical data format [70]. The remaining datasets [71–75] use the NIfTI format for CT scans and, when available, segmentation images.

### Imaging software evaluation

Although the following software met the inclusion criteria, CollectiveMinds (www.cmrad.com) was excluded since it resulted a Web platform for collaborative reporting, accessible only to licensed medical doctors and Papaya (http://mangoviewer.com/papaya.html) was excluded since it was based on the same API of Mango. Finally, ten software tools resulted from the quasi-systematic selection, as listed in Table 5.

**Table 5:** Evaluation of DICOM viewers included in the study

Software	DE-ID	DICOM-SEG		DICOM-SR		License	Release date	Link
		Reading	Writing	Reading	Writing			
RadiAnt	N	N^*^	N	Y	N	Free trial + commercial	29/04/2020	https://www.radiantviewer.com/
ProSurgical3D	N	N	N	N	N	Free trial + commercial	25/06/2019	https://www.stratovan.com/products/pro-surgical-3d
PostDicom	N	Y	N	Y	N	Free trial + commercial	N/A	https://www.postdicom.com/
Horos Viewer	Y	N	N	Y	N	LGPL-3.0	19/12/2019	https://horosproject.org/
3D Slicer	P	Y	Y**	Y**	N	BSD-style	22/05/2019	https://www.slicer.org/
Mango	P	N	N	N	N	RII-UTHSCSA	24/03/2019	http://ric.uthscsa.edu/mango/
ITK-SNAP	N	N	N	N	N	GNU General Public License	12/06/2019	http://www.itksnap.org/pmwiki/pmwiki.php
mEDinria	N	N	N	N	N	BSD 4-Clause	11/06/2020	
mricROgl	Y	N	N	N	N	BSD 2-Clause	31/3/2020	https://www.nitrc.org/projects/mricrogl
BrainVISA Anatomist	N	N	N	N	N	CeCILL License V 2	25/09/2018	http://brainvisa.info/web/index.html

Y, yes; N, no; P, partial

^*^DICOM-SEG successfully loaded but misinterpreted by the software

^**^Including “QuantitativeReporting” extension (https://qiicr.gitbook.io/quantitativereporting-guide/)

All software tools proved to be suitable in basic operations such as loading, selecting, viewing and manipulating the raw images from the probe datasets.

The third and fourth columns of Table 5 show the results, in dichotomous representation, of the software evaluation with the respect to annotation/segmentation and structured report, respectively.

For each selected software tool, the aforementioned procedures were tested for both reading and writing operations. The following results summarize the software evaluation procedure:

- Four out of ten analyzed software tools were able to modify DICOM header by editing DICOM tag values.
- None of the selected software tools allows the writing of regions of interest in the DICOM-SEG format; at the same time, two software tools (PostDicom and 3D Slicer) allow DICOM-SEG reading.
- Four software tools (Radiant, PostDicom and Horos Viewer) allow the reading of information encoded in the DICOM-SR format, and none of the analyzed software tools allows DICOM-SR writing.

Of note, 3D Slicer software allows both writing DICOM-SEG and reading DICOM-SR images by the use of “QuantitativeReporting” extension (https://qiicr.gitbook.io/quantitativereporting-guide/).

In addition, only two software tools (Horos Viewer and MricROgl) allow the de-identification of DICOM folders in full compliance with the DICOM standard.

## Discussion

In the era of big data, it is increasingly important to pay attention and care to data management in order to fully exploit the potential of modern analytical techniques. The definition and the proper use of standards certainly play a leading role in this perspective.

The DICOM standard has been described and evaluated for a series of key actions involved in the radiological workflow. The results of our research first show that the DICOM format fully supports them, allowing to encapsulate in a single format much of the information necessary for subsequent analytical phases.

Considering a typical radiomic workflow [98, 99], an artificial system can find appropriately de-identified data, information related to the patient's clinical status (DICOM-SR) and information on the localization of the region of interest within a single DICOM folder. For example, the details concerning the ROI (DICOM-SEG) can be useful to calculate the radiomic descriptors, thus favoring the aggregation of suitable data to develop reliable systems for classification or prediction of clinical outcomes.

It is interesting to note that although the DICOM format has considerable potential to foster big data analytics, it is only partially exploited in the sharing of imaging data for research purposes.

The results of the imaging database evaluation show that the DICOM-SR format is rarely used to contain clinical information. It should be pointed out that all the 24 datasets providing clinical reports released such information in DICOM-SR format according to guidelines drawn up for challenge tasks or specific initiatives [83, 91, 92, 96]. Moreover, regarding DICOM-SEG, one-third of the analyzed datasets use different and not fully standardized formats to share information on regions of interest. On this topic, the “DICOM4QI” initiative is worthy of note; it aims at evaluating interoperability of the image analysis tools and workstations, applied to exchange of the quantitative image analysis results using DICOM standard [7, 9, 10].

The imaging software evaluation shows that the support provided by software to fully exploit the potential of the DICOM format is still considerably limited, reducing the possibility for researchers and clinicians to create and make available suitable DICOM datasets. Therefore, there is a need to spur the development of initiatives that increase the attention on radiological software not only for the visualization reporting, but also for preparing datasets suitable for big data analytics. Interesting and helpful initiatives, such as OHIF viewer initiative [103] and NCI Imaging Data Commons (IDC), should be highlighted. Although not included in the present study, the first aimed to deliver an extensible platform to support site-specific workflows and accommodate evolving research requirements, according to DICOM specifications. Instead, IDC highlighted the role of the DICOM format as a cornerstone for sharing data and harmonizing analyses [104].

The need to promote the DICOM standard is additionally demonstrated by the analysis of the CT COVID-19 datasets. Indeed, only the two most recent COVID-19 collections released CT data in DICOM format. Although DICOM is the format used in the clinical acquisition routine, its limited adoption may indicate that the actual emergency conditions enhance the difficulty in finding adequate tools to manage the standard and, therefore, to promptly proceed to the de-identification, sharing and care of data. This difficulty may therefore lead researchers to use alternative formats easier to manage and disseminate, such as textual tables and non-medical image data.

It is important to note that this work includes databases mainly oriented to oncological studies, although this was not a prerequisite. Indeed, neurological studies, in which diagnostic images play a decisive role [105–107], have not been evaluated due to the definition of ad hoc standards and tools to manage and share neuroimaging data. In this field, the dedicated standard named Brain Imaging Data Structure (BIDS) [108] has been developed to organize and describe neuroimaging data with the use of different file formats than DICOM (e.g., NIfTI, JSON, text files). Similar to the DICOM, the BIDS standard allows to manage both metadata and derived quantitative measurements, opening to automated data analysis workflows.

It should be considered that, to enhance the feasibility of this work, narrow inclusion criteria that could not allow a comprehensive analysis were chosen and, therefore, it is not possible to exclude that some useful data have been neglected. For example, software tools and databases whose release requires the submission and approval of specific research projects were not considered.

## Conclusions

In conclusion, this work provides an overview of the potential, not always exploited, of the DICOM format for capitalizing the radiological workflow from a big data perspective. The analysis of both the databases and the software shows that further efforts are needed by researchers, clinicians and companies to promote and facilitate the use of standards to increase the value of imaging data, according to FAIR principles.

## Data Availability

This work is based on data and information already available with open access.

## Abbreviations

A/S: Annotations/segmentations
AI: Artificial intelligence
CI: Clinical info
COVID-19: Coronavirus disease 2019
CR: Clinical report
CR: Computed radiography
CSV: Comma separated value
CT: Computed tomography
DICOM: Digital Imaging and Communications in Medicine
DICOM-RT: DICOM radiation therapy
JSON: JavaScript Object Notation
mhd: MetaImage
MR: Magnetic resonance
NEMA: National Electrical Manufacturers Association
NIfTI: Neuroimaging Informatics Technology Initiative
NRRD: Nearly raw raster data
PT (or PET): Positron emission tomography
ROI: Region of interest
RTDOSE: DICOM-RT dose
RTPLAN: DICOM-RT plan
SR: Structured report
TCIA: The Cancer Imaging Archive
TID: Template identifier
XLS: Excel spreadsheet
XML: Extensible markup language
